# 1241. In Vivo Efficacy of Meropenem Against Metallo-ß-Lactamase (MBL)-Harboring *Pseudomonas aeruginosa* and Correlation to In Vitro Susceptibility Upon Addition of EDTA

**DOI:** 10.1093/ofid/ofab466.1433

**Published:** 2021-12-04

**Authors:** Abigail K Kois, Tomefa E Asempa, David P Nicolau

**Affiliations:** 1 Hartford Hospital, Hartford, Connecticut; 2 CAIRD, Hartford Hospital, Hartford, Connecticut

## Abstract

**Background:**

Prior investigations evaluating the predictive value of zinc-depleted media for MBL-susceptibility testing have focused on *Enterobacterales*. Therein, bacterial killing observed with meropenem (MEM) *in vivo* was concordant with its pharmacodynamic profile using MIC values determined in zinc-depleted media compared with conventional cation-adjusted Mueller-Hinton broth (CAMHB). This study aims to evaluate the exposure-response relationship of MEM against VIM- and NDM-harboring *P. aeruginosa* (PSA) using the murine thigh infection model and zinc-depleted MICs.

**Methods:**

MBL-harboring PSA isolates (VIM n=11; NDM n=10) were tested both *in vivo* (neutropenic murine thigh infection model) and *in vitro* (broth microdilution). The 24h murine thigh study was conducted with treatment groups receiving a humanized MEM 2g q8h (3h infusion) dose. Six different zinc-limited media were prepared by the addition of EDTA at concentrations ranging from 3 to 300 mg/L to CAMHB. MEM MICs were determined in triplicate in conventional CAMHB and zinc-limited media. Time > MIC values (generated in each zinc-depleted media) were then plotted against the change in 24h bacterial density count in an Emax model.

**Results:**

Average 0 h bacterial densities were 5.21 ± 0.40 and 5.13 ± 0.81 log_10_ CFU/thigh for NDM and VIM isolates, respectively. MEM resulted in -0.09 CFU reduction to +3.69 CFU growth against NDM isolates. MEM resulted in -2.59 CFU reduction to +4.81 CFU growth against VIM isolates. All MEM MICs in conventional CAMHB were >64 µg/mL for NDM and ranged from 8 to >64 µg/mL for VIM isolates. Increasing EDTA concentrations resulted in several-fold MIC reductions and on average, a larger magnitude of reduction was observed among VIM- (6-fold) compared with NDM-harboring PSA (4-fold) in CAMHB-EDTA 300 mg/L relative to CAMHB. For both NDM- and VIM-harboring PSA, an Emax model with MICs generated in CAMHB+EDTA 30 mg/L (r^2^ = 0.88) provided the highest correlation with MEM *in vivo* activity compared with CAMHB (r^2^ = 0.55).

**Conclusion:**

Results indicate that MIC values generated in conventional CAMHB do not appropriately characterize the *in vivo* efficacy of meropenem against MBL-harboring PSA, and addition of EDTA (30 mg/L) to CAMHB appears to be a viable option for *in vitro* testing of these organisms.

**Disclosures:**

**David P. Nicolau, PharmD**, **Abbvie, Cepheid, Merck, Paratek, Pfizer, Wockhardt, Shionogi, Tetraphase** (Other Financial or Material Support, I have been a consultant, speakers bureau member, or have received research funding from the above listed companies.)

